# Nociceptin/Orphanin Fq in inflammation and remodeling of the small airways in experimental model of airway hyperresponsiveness

**DOI:** 10.14814/phy2.13906

**Published:** 2018-10-28

**Authors:** Gioia Tartaglione, Giuseppe Spaziano, Manuela Sgambato, Teresa Palmira Russo, Angela Liparulo, Renata Esposito, Salvatore Mirra, Rosanna Filosa, Fiorentina Roviezzo, Francesca Polverino, Bruno D'Agostino

**Affiliations:** ^1^ Department of Experimental Medicine School of Medicine Section of Pharmacology University of Campania “L. Vanvitelli” Naples Italy; ^2^ Department of Pharmacy University of Naples Federico II Naples Italy; ^3^ Brigham and Women's Hospital Harvard Medical School Boston Massachusetts

**Keywords:** Airway remodeling, Asthma, N/OFQ, Small airways

## Abstract

It is widely recognized that airway inflammation and remodeling play a key role not only in the central airway but also small airway pathology during asthma. Nociceptin/Orphanin FQ (N/OFQ), an endogenous peptide, and its receptor N/OFQ peptide (NOP) are involved in airway hyperresponsiveness (AHR). We studied a murine model of AHR in order to understand the role of N/OFQ in the inflammation and remodeling of the small airways. Balb/c mice were sensitized to ovalbumin (OVA). At days 0 and 7 (pre‐OVA sensitization) or from day 21 to 23 (post‐OVA sensitization), the mice were treated intraperitoneally with N/OFQ or saline solution. After the last OVA challenge, all OVA‐sensitized mice were aerosol‐challenged with 1% OVA in PBS for 48 h, and then euthanized. Small airway compliance (sC_aw_) was measured and lung samples were collected for histological and molecular evaluations such as perimeter and diameter of small airway, total wall area, airway smooth muscle (ASM) thickness and number of alveolar attachments. Both pre‐ and post‐OVA sensitization N/OFQ treatments induced: (1) increases in sC_aw_; (2) reduction of the bronchial wall thickness; (3) attenuation of the hyperplastic phase of airway smooth muscle mass; and (4) protection against loss of alveolar attachments compared with saline solution treatments. These results suggest that N/OFQ protects against inflammation, and mechanical damage and remodeling of small airways caused by OVA sensitization, suggesting a new potential therapeutic target for asthma.

## Introduction

Asthma is a chronic lung disease characterized by inflammation and remodeling of the airways, recurrent symptoms of reversible airflow obstruction, bronchial hyperresponsiveness, airway inflammation as well as coughing, wheezing, dyspnea, and chest tightness (Brightling et al. 2012; Olin and Wechsler 2014; Nigro et al. 2015). During the asthmatic process, changes in the structure of the airways occur, including subepithelial fibrosis, wall thickening, and hypertrophy and hyperplasia of smooth muscle (Sera et al. 2007; Chen et al. 2010; Urbanek et al. 2016). Similar changes occur not only in the large, but also in the small airways, suggesting that these changes together with persistent inflammation may compromise the lung conduction system by altering mechanical properties of the airways (Macklem 1998; Tashkin 2002; Chen et al. 2010).

Human small airways consist of membranous, terminal, and respiratory bronchi of less than 2 mm diameter (Cottini et al. 2015). In mice, small airways are defined as those having a diameter between 0 and 400 *μ*m (Liu et al. 2006; Sera et al. 2007). Several studies conducted over three decades ago demonstrated that the small airways are pathways of low resistance, contributing to less than 10% of the total resistance to airflow (Cottini et al. 2015). For this reason, the small airways were originally described as the “quiet zone” of the lungs (Harrison et al. 2008; Carr et al. 2017).

Instead, more recent studies (Usmani and Barnes 2012; Usmani et al. 2016) have shown that the small airways are the major site of total lung resistance in patients with moderate‐to‐severe asthma when compared to healthy subjects or to patients with mild asthma (Yanai et al. 1992).

The small airways and the lung parenchyma produce many T helper 2 (Th2)‐related cytokines and chemokines involved in initiation and progression of the asthmatic process (Gallelli et al. 2003; Hamid 2012). N/OFQ is an endogenous peptide activator of the N/OFQ peptide (NOP) receptor, classified as a nonopioid member of the opioid family. The N/OFQ‐NOP receptor system has a dual action in pain processing, consisting of anti‐opioid functions supraspinnaly, and antinociceptive actions in the spinal cord (Singh et al. 2016). In addition, the N/OFQ‐NOP receptor system modulates many physiologic responses, such as anxiety, food intake, learning, locomotor, cardiovascular and renal functions including immunomodulatory function, and effects on isolated airway tissues (D'Agostino et al. 2002). We have previously shown that the N/OFQ and its receptor are implicated in airway hyperresponsiveness (AHR) (Singh et al. 2013, 2016; Sullo et al. 2013) suggesting its potential involvement in the regulation of airway inflammation (D'Agostino et al. 2002, 2005, 2010; Rouget et al. 2004). Thus, we have now evaluated the role of the N/OFQ‐NOP receptor system in the pathogenesis of obstruction, inflammation, and remodeling of the small airways in a conventional murine model of AHR.

## Materials and Methods

### Animal studies

Animal studies reported are in compliance with the arrive guidelines (Kilkenny et al., 2010; McGrath and Lilley, 2015). The investigation was approved by the Veterinary Animal Care and Use Committee of the Second University of Naples (1966/7.17.2012) and conforms to the National Ethical Guidelines of the Italian Ministry of Health and the Guide for the Care and Use of Laboratory Animals (National Institute of Health, Bethesda, MD, USA, revised 1996). All experimental procedures were in accordance with Italian D.Lgs 26/2014, application of the EU Directive 2010/63/EU. BALB/c mice were obtained from Harlan Laboratory (Udine, Italy). Mice were housed in the animal facility of the University of Campania “L.Vanvitelli”, in standard conditions. Food and water were supplied ad libitum. Room temperature was 22–24°C, relative humidity was 40–50%, and the day/night cycle was set at 12 h/12 h. Mice were acclimatized for 1 week before starting any procedures in five mice per cage.

### Experimental protocol

**Figure 1:**
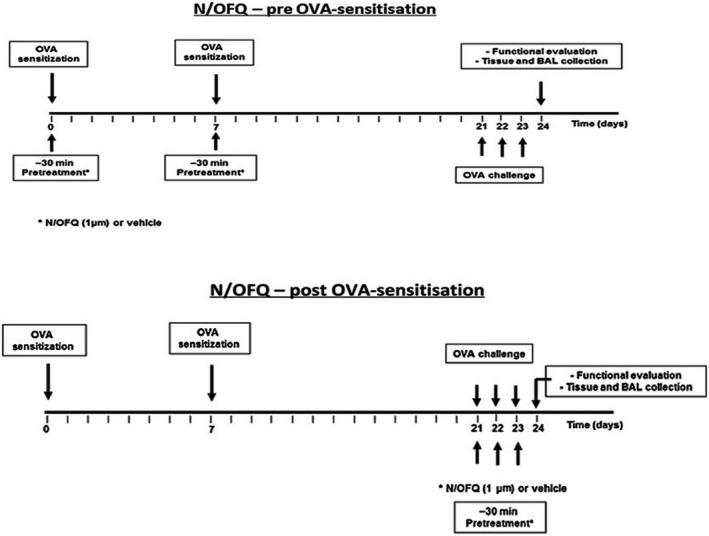
Experimental protocols. Pre‐OVA sensitization (A) and post‐OVA sensitization (B) treatments with N/OFQ.

Female BALB/c mice at 6 weeks of age were used in this study. Mice were randomized into five experimental groups: naïve, OVA alone (OVA), N/OFQ alone (N/OFQ), pre‐treatment with N/OFQ followed by OVA challenge (OVA + N/OFQ pre), and OVA challenge followed by N/OFQ treatment (OVA + N/OFQ post), (*n* = 6 for each group). Animals were sensitized to allergen ovalbumin (OVA, Sigma‐Aldrich, St. Louis, MO, USA) by s.c. injection with 0.4 mL of 10 *μ*g OVA, absorbed to 3.3 mg of aluminium hydroxide gel in sterile saline solution at days 0 and 7. From day 21 to 23, all OVA‐sensitized mice were aerosol challenged (7‐min‐long daily sessions) with 1% OVA in PBS using an ultrasonic nebulizer (De Vilbiss Health Care, UK Ltd., Heston, Middlesex, UK).Two different experimental protocols were used for N/OFQ treatment. In the first protocol (pre‐OVA sensitization), vehicle or N/OFQ was administered i.p., at day 0 and 7, 30 min before each allergen injection (Fig. 1A). In the second protocol (post‐OVA sensitization), vehicle or N/OFQ was administered i.p. from day 21 to 23, 30 min before each OVA aerosol challenge (Fig. 1B). Twenty‐four hours after the last aerosol challenge, animals were killed, bronchopulmonary function was performed, and pulmonary tissues were collected. In an independent experiment, using the same experimental protocol, bronchoalveolar lavage (BAL) fluid collection was performed.

### Airway responsiveness

Lung reactivity was assessed in an isolated and perfused mouse lung model (IPL‐1), Hugo Sachs Elektronik – Harvard Apparatus, as described in details in Spaziano et al. (2016) (Singh et al. 2013). Mice were anesthetized with ketamine HCl 40 mg kg^‐1^ i.p. and medetomidine hydrochloride 0.15 mg kg^‐1^ i.p. The skin was incised from the abdomen to the throat, and the trachea was exposed with a small incision and cannulated. The abdomen was opened, the diaphragm resected, and 50 *μ*L of heparin injected into the heart. The mouse was exsanguinated after incision of the renal vena, the thorax was opened, and the two thoracic halves were fixed at the sides of the cork plate with two cannulas. At this point the pulmonary artery was cannulated through a small piece of skin on the right atrium, so that the arterial cannula was inserted into the pulmonary artery and fixed by the ligature. Drugs were administered through the arterial cannula into the pulmonary artery. After preparation, the lungs were perfused in a nonrecirculating fashion through the pulmonary artery at a constant flow of 1 mL/min, resulting in a pulmonary artery pressure of 2–3 cmH_2_O. As a perfusion medium we used RPMI 1640 medium lacking phenol red (37°C) that contained 4% low‐endotoxin‐grade albumin. The lungs were ventilated by negative pressure (−3 to −9 cmH_2_O) with 90 breaths/min and a tidal volume of ∼200 *μ*L. Every 5 min a hyperinflation (−20 cmH_2_O) was performed. Artificial thorax chamber pressure was measured with a differential pressure transducer (Validyne DP 45‐24) and air flow velocity with a pneumotachograph tube connected to a differential pressure transducer (Validyne DP 45‐15). The lungs respired humidified air. The arterial pressure was continuously monitored by means of a pressure transducer (Isotec Healthdyne) that was connected with the cannula ending in the pulmonary artery. All data were transmitted to a computer and analyzed by the Pulmodyn software (Hugo Sachs Elektronik, March Hugstetten, Germany). For lung mechanics, the data were analyzed by applying the following formula: *P*  =  *V*·*C* ^−1^ + *r*l·dV·d*t* ^−1^, where *P* is chamber pressure, *C* pulmonary compliance, *V* tidal volume, and *r*l airway resistance. The machine was set up such that, after 60 min, the mean tidal volume was 0.21 ± 0.02 mL (*n* = 61), the mean airway resistance 0.23 ± 0.08 cmH_2_O·s·mL^−1^, and the mean pulmonary artery pressure 2.9 ± 1.4 cmH_2_O. The measured airway resistance was corrected for the resistance of the pneumotachometer and the tracheal cannula of 0.6 cmH_2_O·s·mL^−1^. Successively, a repetitive dose‐response curve to acetylcholine (Ach, Sigma‐Aldrich, St. Louis, MO, USA, 10^−8^ to 10^−3^ mol/L) in all experimental groups was obtained. The five experimental groups, naïve, OVA alone (OVA), N/OFQ alone (N/OFQ), pre‐treatment with N/OFQ followed by OVA challenge (OVA + N/OFQ pre), and OVA challenge followed by N/OFQ treatment (OVA + N/OFQ post) were analyzed as follows.

### Bronchoalveolar lavage (BAL)

Mice were anesthetized with ketamine HCl 40 mg kg^−1^ i.p. and medetomidine hydrochloride 0.15 mg kg^−1^ i.p and then euthanized. Mouse BAL fluid was collected as follows: 1.5 mL of saline was instilled and withdrawn from the lungs via an intratracheal cannula; this lavage was performed three times, and different samples were collected. BAL fluid was centrifuged at 1000*g* for 10 min at 4°C. The supernatant was transferred into tubes and stored at −80°C before use to analyze the cytokine production. Cell pellets were resuspended in PBS to a final volume of 0.5 mL for total and differential cell counting. Total cell count was performed using the Countess automated cell counter (Invitrogen). Differential counting was performed on Diff Quik (Reagena, Gentaur, Italy) stained cytospin.

### Total and differential cell count

Total cell count was performed using the Countess automated cell counter (Invitrogen), which evaluates cell number and viability using trypan blue stain according to the manufacturer's instructions. Differential counting was performed on Diff‐Quik (Reagena, Gentaur, Italy) stained cytospin. At least 200 cells were counted on each cytospin according to standard morphological criteria under light microscopy.

### Histology

Lung specimens obtained from mice pre‐treated with N/OFQ 30 min before OVA challenge and mice challenged with OVA without pre‐treatment (untreated controls) were examined. Lungs were removed 24 h after the OVA challenge, fixed in 4% formaldehyde in phosphate‐buffered saline (PBS) at pH 7.2 and, after dehydration through an alcohol series, were embedded in paraffin wax. Sections (4–5 mm thick) were cut from each block and stained with hematoxylin–eosin.

For each animal, at least five small size airways (diameter > 400 μm) were identified. These airways had to be intact, transversally cut and should not contain cartilage or glands. In each airway, the internal perimeter along the subepithelial basement membrane and the luminal diameter in a plane perpendicular to the long axis of the lumen (D) were measured. Morphometric analysis of airway area and smooth muscle area was performed with ImageJ software. Total wall area, everything between basement membrane and external wall border, and the smooth muscle area were measured and normalized for airway perimeter in each airway for comparative analyses and correlations (Sera et al. 2007; Plant et al. 2012). Alveolar attachments (AA), the alveolar septa that extend radially from the outer wall of nonrespiratory bronchioles, were counted over the external circumference. The results were expressed as percent abnormal attachments (% abnormal = [number of abnormal (no. attached + number of abnormal)] × 100), for each animal (Saetta et al. 1985).

Any AA showing discontinuity from the peribronchial layer or rupture was denoted as a destroyed attachment.

The numbers of intact alveolar attachments per millimeter of airway external perimeter and destroyed alveolar attachments were calculated. Samples were analyzed with a Leica DM 5000B microscope a Zeiss LSM 700 confocal microscope at magnification 20X. All histological analysis were performed using the ImageJ software (National Institutes of Health, Bethesda, MD).

### Histochemistry and immunofluorescence

For immunofluorescence, after deparaffinization and rehydration, tissue sections were treated with 10% normal donkey serum for 30 min at room temperature and then incubated with the primary antibodies diluted in PBS. After, the sections were incubated with the FITC conjugated and tetramethylrhodamine isothiocyanate conjugated secondary antibodies (Jackson Immuno Research). Nuclei were stained with DAPI. For the assessment of inflammation, sections were stained with hematoxylin–eosin (HE). The number of eosinophils mm^−2^ of the peribronchial tissue was measured. The number of mast cells mm^−2^ of the lung tissue was measured after staining with toluidine blue. Mucin production was assessed by immunolabeling with anti‐mucin5 AC antibody (Abcam, Cambridge Science Park, UK). Mucin‐positive cells were quantified in the epithelial layer of the bronchi by counting labeled cells per total number of cells within the airway epithelium. All samples were analyzed with a Leica fluorescence microscope (Leica Microsystems GmbH, Wetzlar, Germany) and a Zeiss LSM 700 confocal microscope (Carl Zeiss Microscopy GmbH, Jena, Germany). The values of corrected total fluorescence of N/OFQ per unit area of a peribronchial tissue from control and OVA mice were obtained using IMAGEJ software (imagej.nih.gov).

### Statistical analysis

Results are reported as mean ± SEM. Significance for multiple comparisons was determined by one‐way ANOVA and Bonferroni's posttest. Lung reactivity curves were compared using a two‐way ANOVA followed by Bonferroni posttest. A value of *P* < 0.05 was considered as significant.

## Results

### Functional and cell count evaluations

Small airway obstruction has important physiological and clinical implications in asthma. Thus, it is crucial to evaluate the small airway compliance (sC_aw_) in order to define the overall respiratory function in asthma.

**Figure 2:**
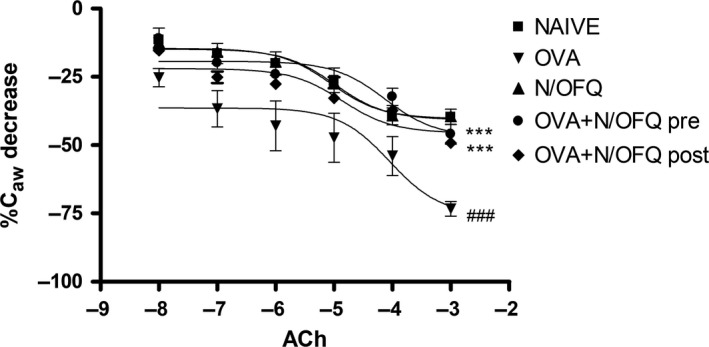
Measurements of airway compliance. Mice treated with N/OFQ show an increase in airway compliance compared to untreated OVA‐sensitized group. Data in naive, OVA alone, N/OFQ alone, pre‐ and post‐ OVA sensitization N/OFQ‐treated groups are represented mean ± SEM. * *P *<* *0.001 versus OVA; ^#^ *P *<* *0.001 versus naïve.

Untreated OVA‐challenged mice had increased ACh‐induced bronchoconstriction, and significantly lower airway compliance than naïve mice. N/OFQ treatments caused a significant increase in sC_aw_ compared with mice treated with saline (Fig.**** 2), indicating that N/OFQ was able to partially restore the normal bronchial reactivity of the small airways.

In line with our previous studies (Singh et al. 2016), the analysis of BAL fluid samples showed a significant increase in total eosinophil and lymphocyte number in untreated OVA‐challenged mice compared to control group. The increase in total eosinophil and lymphocyte number in untreated OVA‐challenged mice was significantly reduced by N/OFQ treatment (data not shown.

### Histology of small airways

The accelerated decline of respiratory flow in asthmatic subjects is accompanied by remodeling of parenchyma and airways. Thus, we conducted a histological evaluation of the parenchymal and airway tissues.

**Figure 3:**
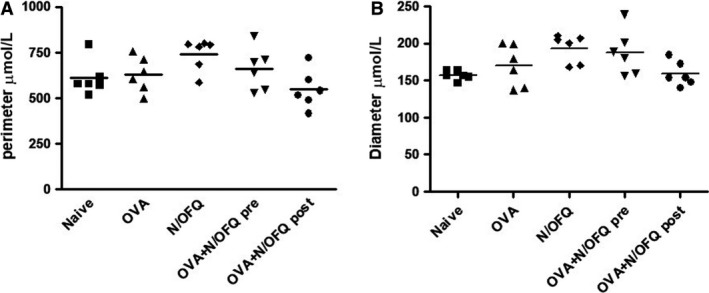
Measurements of perimeter (A) and diameter (B) of the small airways. N/OFQ treatments do not alter significantly the perimeter and diameter of the small airways. Data in naive, OVA alone, N/OFQ alone, pre‐ and post‐ OVA sensitization N/OFQ‐treated groups are represented as mean ± SEM.

Small airways with a short‐to‐long diameter ratio of less than one‐third were excluded to avoid measurements in tangentially cut airways. The airway perimeter was similar in mice treated with N/OFQ and in control mice (Fig. 3A). Similarly, the airway diameter was not significantly different between the five groups of mice examined (Fig. 3B).

### Total wall area

Subepithelial fibrosis in the basement membrane region of the airways is a typical feature of airway remodeling in asthmatic patients. Thus, we further assessed the bronchial wall thickness in the small airways.

The bronchial wall thickness in untreated OVA‐challenged mice was significantly greater than that in the control animals.

**Figure 4:**
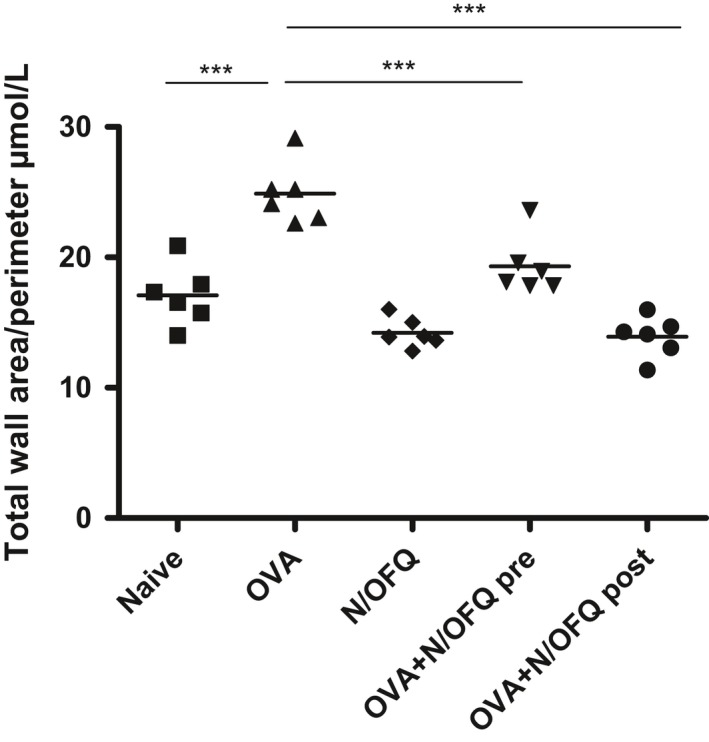
Measurements of wall thickness of the small airways. Both pre‐ and post‐OVA sensitization N/OFQ treatments significantly reduced the total wall area of the small airways compared to OVA‐sensitized mice not treated with N/OFQ. Data in naive, OVA alone, N/OFQ alone, pre‐ and post‐ OVA sensitization N/OFQ‐treated groups are represented as mean ± SEM. *P <* *0.001.

Both pre‐ and post‐OVA sensitization N/OFQ treatments caused a significant reduction of the bronchial wall thickness as compared with the corresponding area in untreated OVA‐challenged mice (Fig.**** 4).

### Airway smooth muscle

**Figure 5:**
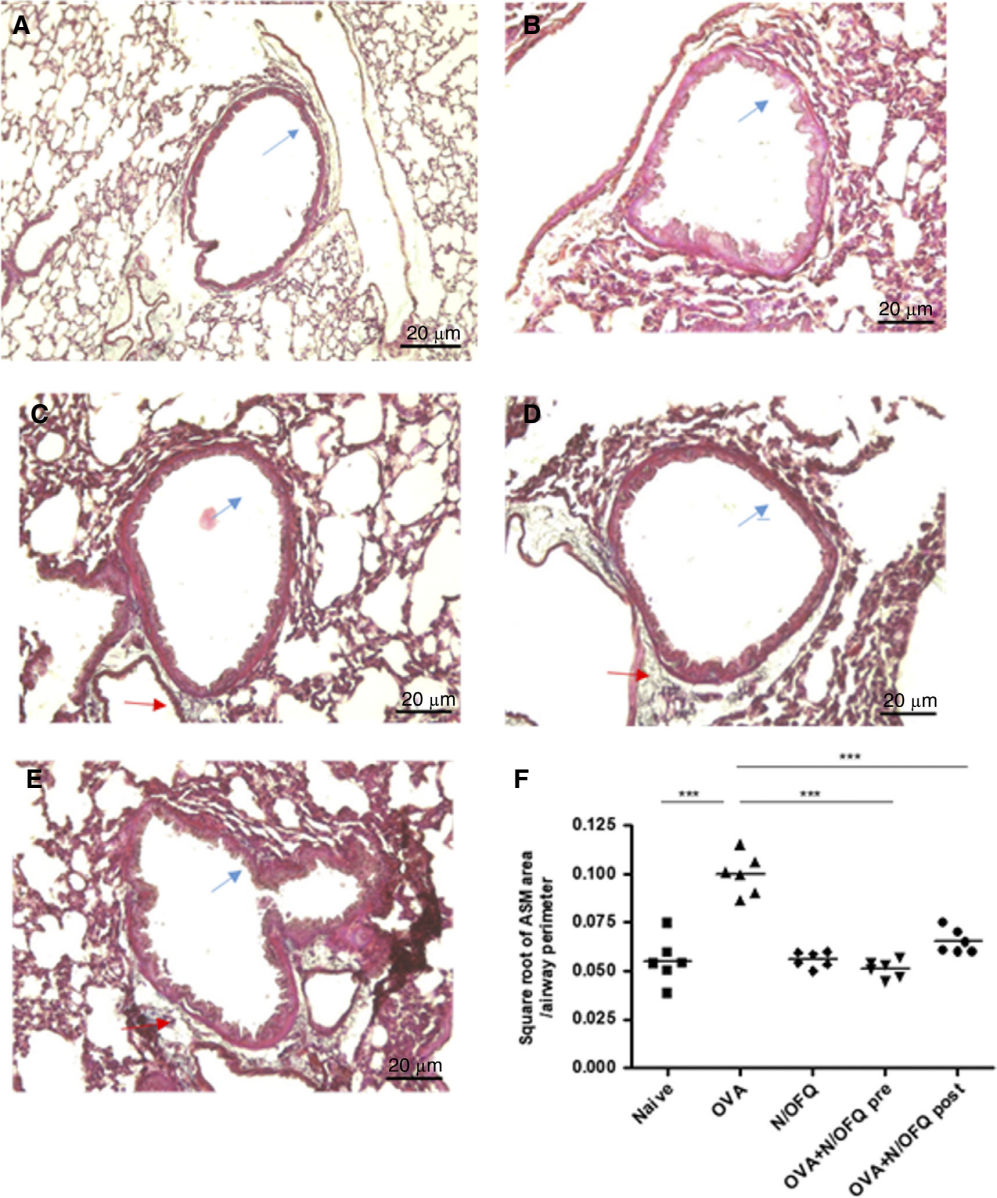
Measurements of airway smooth muscle mass (ASM). In (A naïve, B OVA alone, C N/OFQ alone, D OVA+N/OFQ pre, E OVA+N/OFQ post) representative pictures of Trichrome‐stained lung sections in the 5 experimental groups are shown. Blu Arrows indicate bronchial wall thickening; red arrows indicate airway remodeling. (F) shows the quantification of airway smooth muscle mass (ASM) in the five experimental groups. N/OFQ treatment attenuated the increase of airway smooth muscle mass observed in OVA‐sensitized mice not treated with N/OFQ. Data in naive, OVA alone, N/OFQ alone, pre‐ and post‐ OVA sensitization N/OFQ‐treated groups are represented as mean ± SEM. *P <* *0.001.

We assessed the structural changes of the airway smooth muscle (ASM), an important factor determining the mechanical properties of the small airways. Morphometric analysis showed an increase of ASM in untreated OVA‐challenged mice compared to naïve group. Conversely, both pre‐ and post‐OVA sensitization N/OFQ treatments protected against the increase of airway smooth muscle mass observed in the OVA‐challenged group, indicating that N/OFQ treatment reduced the hyperplastic phase of ASM (Fig.**** 5A–F).

### Alveolar attachments

Changes to the small airways could also be associated with loss of alveolar attachments.

**Figure 6:**
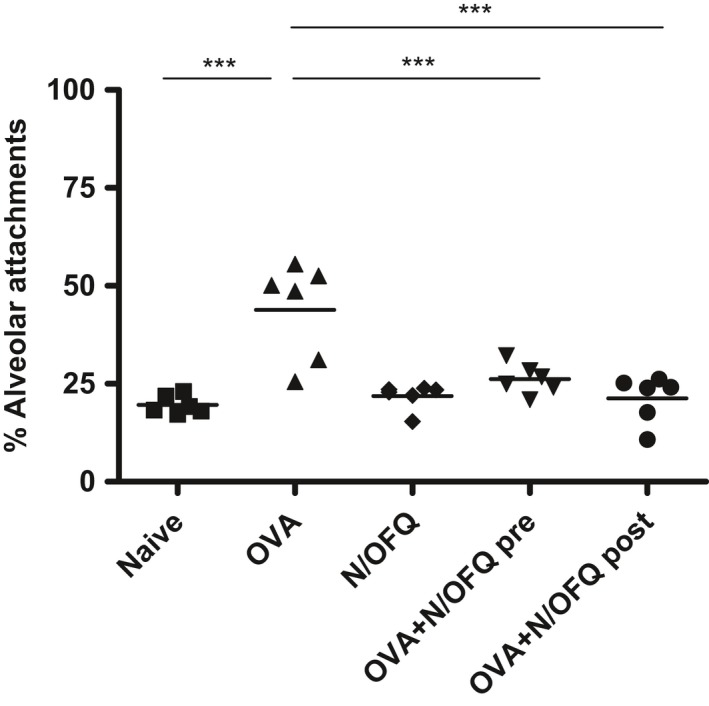
Quantification of alveolar attachments in the five experimental groups. Both pre‐ and post‐OVA sensitization N/OFQ treatments significantly restored alveolar attachments. Data in naive, OVA alone, N/OFQ alone, pre‐ and post‐ OVA sensitization N/OFQ‐treated groups are represented as mean ± SEM. *P <* *0.001.

Both pre‐ and post‐OVA sensitization N/OFQ treatments protected against the loss of alveolar attachments induced by OVA sensitization (Fig.**** 6), suggesting an important role of N/OFQ in the remodeling of the small airways.

## Discussion

It is well established that large airways are primarily involved by the inflammatory process in asthma. However, histopathological evidence from several studies has clearly shown that the inflammation in asthmatic subjects also involves the small airways (Wagner et al. 1998). N/OFQ protects against bronchial hyperresponsiveness and has immunomodulatory properties in the lung (Saetta et al. 1985; Wagner et al. 1998; Corsico et al. 2003; Sullo et al. 2013; Spaziano et al. 2016) but its effects during asthmatic airway hyperresponsiveness have not been clarified yet. Thus, we studied the role of the N/OFQ peptide in the inflammatory and remodeling process of the small airways, by using an ex vivo histological approach. We now show for the first time that N/OFQ is crucial in regulating the mechanical properties and the remodeling of the small airways.

In many respiratory diseases such as asthma, COPD, interstitial lung diseases and obliterative bronchiolitis, significant compliance abnormalities occur, mainly in localized regions of bronchi and bronchioles (Sera et al. 2013). In particular, changes in airway distension and compliance during airway hyperresponsiveness are predominantly due to perturbations of the small airways (Sera et al. 2007, 2013). In our study, we tested both prophylactic and therapeutic approaches by administering N/OFQ treatment prior to (prophylactic approach) or after (therapeutic approach) OVA sensitization. Both approaches led to significantly decrease of sC_aw_ compared to naïve group suggesting that N/OFQ could represent a potential therapeutic target for asthma both in prophylaxis and control of acute symptoms.

Inflammation is thought to be the main cause of the increased inner airway wall thickness in small airways in COPD and asthma (Jeffery 2004; Spaziano et al. 2017). In the current study, the BAL analysis and histological evaluations of lung tissue confirmed our previous observations (Singh et al. 2016) indicating that N/OFQ has protective effects against OVA‐induced lung inflammation and peribronchial inflammatory infiltrates. Airway perimeter and diameter were similar in all the experimental groups studied. This is not surprising, as diameter and perimeter are considered to be reliable markers of airway size that remain constant even during bronchoconstriction, as well as smooth muscle tone and lung volume.

Both pre‐ and post‐OVA sensitization N/OFQ treatments led to a significant reduction in the area of the bronchial wall compared with mice treated with saline. Changes in the thickness of the airway wall have been documented in respiratory tracts of less than 2 mm diameter suggesting that the small airway wall might undergo morphological changes during asthma (Roche 1998; Jeffery 2004). We here show that both pre‐ and post‐OVA sensitization N/OFQ treatments were associated with a reduction of the hyperplastic phase of ASM. The relative contribution of ASM in small airways is greater than that in the large airways. In fact, previous studies failed to detect any major change in the large respiratory tract of smooth muscle mass during asthma (Gallelli et al. 2003).

Finally, morphological changes of the small airways are associated with perturbed alveolar attachments (Saetta et al. 1994; Harrison et al. 2008). Destruction of the alveolar attachments to bronchiolar walls leads to a reduction in the caliber of the airways because of loss of radial traction forces (Mauad et al. 2014; Chen et al. 2010). Destruction and therefore loss of alveolar attachments are early changes leading to the destruction of the lung parenchyma and loss of elastic recoil (Saetta et al. 1994). Some studies (Harrison et al. 2008; Hamid 2012) have shown an increased number of damaged peribronchial alveolar attachments in fatal asthma, with patients who died of asthma presenting an increased proportion of damaged alveolar attachments when compared with patients without asthma (Saetta et al. 1994; Mauad et al. 2004). Our results indicate the presence of damaged alveolar attachments in OVA‐sensitized mice. Interestingly, both pre‐ and post‐OVA sensitization N/OFQ treatments protected against the loss of alveolar attachments, suggesting a protective role of nociceptin in maintaining the patency of the small airways during asthma.

There are two notable limitations to our study. Firstly, we have not performed mechanistic studies; in fact, we already documented mechanism of action of N/OFQ‐NOP system on large airways in our previous publications. Because, N/OFQ showed similar effects on small airways, the mechanisms are likely the same.

Secondly, the lack of antagonist data; in fact, the utilization of antagonists in several other studies is questionable because the commercially available agonists only show partial agonist activity making the interpretation of the results debatable.

Data in the current manuscript represent an incremental advance over our previous publications, that emphasize and confirm the role of N/OFQ‐NOP in the airways. However, we believe that this study lays the foundation for future studies investigating how N/OFQ‐NOP protects against small airway remodeling in asthma.

In conclusion, our data suggest that the small airways play a major role in the development of airway hyperresponsiveness during asthma. Both prophylactic and therapeutic treatments with N/OFQ protected the mice from OVA‐induced inflammation and from asthma‐related morphological changes such as small airway remodeling and loss of alveolar attachments. Thus, therapies aimed at boosting N/OFQ levels in the lung could have beneficial potentials in the treatment of clinical asthma.

## Conflict of Interest

The authors report no conflicts of interest in this work.
